# Case report: A successfully treated case of community-acquired urinary tract infection due to *Klebsiella aerogenes* in Bangladesh

**DOI:** 10.3389/fmed.2023.1206756

**Published:** 2023-06-26

**Authors:** Razib Mazumder, Arif Hussain, Bithika Bhadra, Jody Phelan, Susana Campino, Taane G. Clark, Dinesh Mondal

**Affiliations:** ^1^Laboratory Sciences and Services Division, International Centre for Diarrhoeal Disease Research, Bangladesh (icddr, b), Dhaka, Bangladesh; ^2^Department of Biochemistry and Microbiology, North South University, Dhaka, Bangladesh; ^3^Department of Infection Biology, London School of Hygiene and Tropical Medicine, London, United Kingdom; ^4^Department of Infectious Disease Epidemiology, London School of Hygiene and Tropical Medicine, London, United Kingdom

**Keywords:** *Klebsiella aerogenes*, community-acquired urinary tract infection, extensive drug resistance (XDR), ESBLs (extended spectrum β-lactamases), type 2 diabetes mellitus, Bangladesh

## Abstract

*Klebsiella aerogenes*, a nosocomial pathogen, is increasingly associated with extensive drug resistance and virulence profiles. It is responsible for high morbidity and mortality. This report describes the first successfully treated case of community-acquired urinary tract infection (UTI) caused by *Klebsiella aerogenes* in an elderly housewife with Type-2 diabetes (T2D) from Dhaka, Bangladesh. The patient was empirically treated with intravenous ceftriaxone (500 mg/8 h). However, she did not respond to the treatment. The urine culture and sensitivity tests, coupled with bacterial whole-genome sequencing (WGS) and analysis, revealed the bacteria to be *K. aerogenes* which was extensively drug-resistant but was susceptible to carbapenems and polymyxins. Based on these findings, meropenem (500 mg/8 h) was administered to the patient, who then responded to the treatment and recovered successfully without having a relapse. This case raises awareness of the importance of diagnosis of not-so-common etiological agents, correct identification of the pathogens, and targeted antibiotic therapy. In conclusion, correctly identifying etiological agents of UTI using WGS approaches that are otherwise difficult to diagnose could help improve the identification of infectious agents and improve the management of infectious diseases.

## 1. Introduction

Community and hospital-acquired urinary tract infections (UTIs) are predicted to afflict approximately 405 million individuals around the globe. It has caused over 0.23 million deaths in 2019, leading to 5.2 million disability-adjusted life years (1). The treatment of UTIs usually consists of broad-spectrum antibiotics. Globally, the incidence of UTIs caused by multidrug-resistant pathogens is rising alarmingly. These infections can also be life-threatening in some cases (2). In 2022, a study of community-acquired urinary tract infections in Bangladesh revealed that *Escherichia coli* was the most prevalent bacterial pathogen, followed by *Streptococcus* Spp., *Klebsiella* Spp., *Enterococcus* Spp., *Pseudomonas* Spp., *Staphylococcus* Spp., *Enterobacter* Spp., *Proteus* Spp., *Acinetobacter* Spp., *Staphylococcus saprophyticus, Staphylococcus aureus, Corynebacterium, and Serratia* (3). A wide variety of bacterial infections may cause UTIs, reinforcing the need for the early identification of the etiological agent to properly manage UTIs (3–5).

There are abundant reports on the prevalence of antibiotic-resistant *Escherichia coli* and *Klebsiella pneumoniae* in UTIs. However, reports on *Klebsiella aerogenes* are limited (6–8). *K. aerogenes* is a gram-negative, facultative anaerobe, motile, rod-shaped, non-spore-forming member of the Enterobacteriaceae family. It was formerly *Enterobacter aerogenes* but was renamed *Klebsiella aerogenes* as it was more closely related to *Klebsiella* species than *Enterobacter* species (9, 10). *K. aerogenes* is a part of the ESKAPE group of pathogens (11) that significantly impact public health. *K. aerogenes* is ubiquitous, a habitant of the human gastrointestinal tract, and a prominent opportunistic pathogen causing nosocomial infections than community-acquired infections. It can cause UTIs, skin and soft tissue and respiratory infections, and bloodstream infections in immune-compromised or patients with damaged intestinal mucosa (12, 13). *K. aerogenes* has been associated with high mortality rates in patients from intensive care units (14).

Community-acquired urinary tract infections account for a large proportion of infectious diseases in the female population worldwide (15). The likelihood of UTIs in women increases with age; ~13% of women aged between 60 and 70 years old experience bacteriuria at least once in their lifetime (16). Urine glucose also promotes bacterial growth and colonization. Consequently, infections in diabetic patients may lead to extended hospital stays, recurrence, renal complications, bloodstream infection and septic shock (17). According to rough estimates, UTIs are fifteen times more likely to occur in diabetic patients, emphasizing the necessity for prompt identification and management of UTIs (18).

Herein, we describe the first successfully treated case of community-acquired UTI due to extensively drug-resistant (XDR) *K. aerogenes* in an elderly housewife suffering from Type-2 diabetes (T2D) in Bangladesh.

## 2. Case presentation

A 63-year-old female housewife from Narayanganj district of Dhaka division in Bangladesh attended the outpatient department of a local private hospital on January 2022. Her main complaints were dysuria, fever with chills, lethargy and mild lower abdominal pain for 6 days. She had a history of hypertension and T2D for 4 years. She was on medication for hypertension [Bisoprolol Hemifumarate (5 mg)] and diabetes [Linagliptin plus Metformin Hydrochloride (2.5 mg plus 500 mg)]. On consultation, the patient was found febrile with a 101°F body temperature, and her pulse, respiratory rate, blood pressure and oxygen saturation were detected to be 109 pulses/min, 20 breaths/min, 128/84 mmHg, and 97% on room air, respectively. She did not have a recent history of a hospital visit, travel, contact with the pet, or ingesting raw food. The patient provided a blood sample for a laboratory investigation. An initial hematological assessment of the blood specimen demonstrated that the patient's hemoglobin was 10.3 g/dl and the erythrocyte sedimentation rate was 50 mm/h. In particular, the number of leukocytes was 13,000 cells/mm^3^, with 81% neutrophils, 26% lymphocytes, 3% eosinophils, and 2% monocytes. The platelet counts, as well as other blood cell variables, were normal. Blood biochemistry analysis showed random blood glucose raised at 10.3 mmol/L and C-reactive protein elevated at 85 mg/ml with normal hepatorenal function parameters. A COVID-19 screening test was done per the hospital requirements and a negative result was obtained (19, 20). Preliminarily, it was suspected to be a case of UTI. Therefore, the patient was empirically treated with intravenous ceftriaxone (500 mg/8 h) for 7 days. However, she did not respond to the treatment and symptoms like increased frequency of painful and burning micturition, fever, and abdominal pain continued while she was admitted to the hospital (Table 1). Therefore, a clean catch midstream urine sample was obtained and sent to the laboratory for routine microscopic examination and microbiological investigation. The urine sample was turbid and revealed the presence of 20 to 30 pus cells per high-power field. The urine sample was inoculated onto 5% sheep blood agar, MacConkey's agar, and CHROMagar Orientation agar plates and incubated the plates at 37°C in an aerobic environment (21). After overnight incubation, a significant (>10^5^ CFU/ml) growth of single-type colonies was observed on all culture plates. The bacteria produced 2–3mm, circular, raised, mucoid, pale pink color colonies on MacConkey agar, gray-white colonies on a blood agar plate with gamma hemolysis and metallic blue pigmented colonies on a CHROMagar Orientation plate. Based on the colony morphology on MacConkey and CHROMagar Orientation agar plates, we suspected it would be either *Klebsiella* or *Enterobacter* species. Gram staining revealed gram-negative bacilli. Further, biochemical characterization was carried out as described previously (22). The bacterium was finally identified as *K. aerogenes* based on its gram-negative morphology, motility, utilization of citrate, decarboxylation of Ornithine, reduction of nitrate and fermentation of glucose and lactose with the production of acid and gas (Table 2) (23). The strain was further tested with the API 20E test (BioMérieux, France), and it confirmed the strain as *Enterobacter aerogenes* with 96% certainty. Antibiotic susceptibility testing followed the Kirby-Bauer disc diffusion method using 21 antibiotic disks representing 11 classes (24–27). The *K. aerogenes* (RD99) strain was resistant to most antibiotic classes tested, including aminoglycosides, β-Lactams (penicillin), β lactams (cephalosporins), amphenicol, fluoroquinolones, folate drugs, tetracyclines, phosphonic acid, and glycycline. However, the strain was susceptible to carbapenems and polymyxins (Table 3).

**Table 1:** Case report timeline with relevant data from the entire episode of care.

**Timeline**	**Relevant data from the case presented**
Day 1 to 6 (2^nd^ to 7^th^ January, 2022)	**Symptoms**: dysuria, fever accompanied by chills, lethargy, and mild lower abdominal pain.
Day 7 (8 January 2022)	**Doctor consultation**: the patient was found febrile with a 101°F body temperature.
Day 7 or 8 (8^th^ or 9^th^ January, 2022)	**Blood test**: revealed elevated blood glucose (10.3 mmol/L) with an elevated C-reactive protein (85 mg/ml). **COVID tests**: was found COVID negative by a RT PCR test.
Day 9 (10 January 2022)	**Empirical treatment**: was initiated with intravenous ceftriaxone (500 mg/8 h) to be continued for 7 days.
Day 15 (16 January 2022)	**Patient response**: The patient did not respond to the treatment, as symptoms persisted despite the administered therapy.
Day 16 (17 January 2022)	**Urine culture, AST and WGS analysis**: A urine sample was sent for urine culture and sensitivity testing and whole-genome sequencing (WGS).
Day 17 to 22 (18^th^ to 23^st^ January, 2022)	**Turnaround time for tests**: microbiological identification, AST, WGS and data analysis.
Day 23 (24 January)	**Evidence-based treatment**: Based on the results of the aforementioned tests, an evidence-based treatment was initiated, and the patient has been prescribed meropenem at a dosage of 500 mg/8 h for 14 days.
Day 30 (31 January)	**Symptoms relieved**: the case became afebrile with no complaints of dysuria and abdominal pain. Moreover, blood and Urine Culture tests were performed and found negative.
Day 36 (6 February 2022)	**Patient recovery**: the patient made a full recovery.
28 February 2022, 30 March 2022, 29 April 2022	**Follow-up tests**: the results of all three-monthly follow-up culture and sensitivity tests were negative, and no relapse of infection was detected.

**Table 2:** Bacteriological and biochemical characteristics of *Klebsiella aerogenes* strain-RD99.

**Tests performed**	**Test result (Strain RD99)**
**Colony morphology**	Medium size, glossy, mucoid colony
**Gram stain**	Gram negative bacilli
**Catalase test**	Positive
**Oxidase test**	Negative
**Kligler's Iron Agar (KIA) test**	
a. Acid production in slant	Positive
b. acid production in butt	Positive
c. Hydrogen sulfide production (H_2_S)	Negative
d. Gas production	Positive
**Motility Indole Ureas Test (MIU)**	
a. Motility	Positive (After 36 h)
b. Indole Production	Negative
c. Urea hydrolysis	Negative
**Simmons citrate reaction test**	Positive
**Acetate**	Negative
**Sugar fermentation**	
a. Glucose	Positive
b. Lactose	Positive
c. Sucrose	Positive
d. Maltose	Positive
e. Mannose	Positive
f. Arabinose	Positive
g. Sorbitol	Positive
h. Mannitol	Positive
i. Inositol	Positive
j. Esculin	Positive
**Nitrate reduction**	Positive
**Gelatine liquefaction**	Negative
**ONPG**	Positive
**Vogas-proskauer**	Positive
**Lysine decarboxylase**	Positive
**Ornithine decarboxylase**	Positive (After 36 hours)
**Arginine Dihydrolase**	Negative
**Haemolysis on Blood Agar**	Gamma-hemolysis
**Growth on agar media**	
a. MacConkey Agar	Light pink color colony
b. SS agar agar	Light pink color colony
c. CHROMagar™ Orientation	Metallic blue color colony
d. Blood agar	Gray white colony
e. Gelatin agar	White colony
**Growth temperature**	26–40°C
**API 20E number**	5305773 (Detect *Enterobacter aerogenes* with 96% pro bability)

**Table 3:** Antibiotic susceptibility, resistome profile, mobilome and plasmid replicons in *Klebseilla aerogenes* (RD99) isolated from Dhaka, Bangladesh.

**Classes**	**Antibiotics**	**Interpretation**	**Antibiotic resistance genes**	**Genome locus (ESBLs gene)**	**MGE*S***	**Mutation in Outer membrane porins**	**Plasmids**
**Aminoglycosides**	**Amikacin (AK)-30** **μg**	**R**	***aph(3′')-Ib, aph(6)-Id, aac(3)-Ile, aac(6′)-Ib-cr***	**Plasmid**		**OmpK36 Mutations A217S N49S D224E L191Q F207W**	**Col440I IncFIB(K) IncFII(K) IncN IncR**
	**Gentamicin (CN)-10** **μg**	**R**					
**β-Lactams (Penicillin)**	**Ampicillin (Amp)-10** **μg**	**R**	***bla*_TEM − 1B_**	**Plasmid**	**Tn2**		
**β** **Lactams (Cephalosporins)**	**Amoxycillin/ Clavulanic Acid (AMC)-30** **μg**	**R**					
	**Cefepime (FEP)-30** **μg**	**R**					
	**Cefixime (CFM)-5** **μg**	**R**	***bla***_**ampC**_	**Chromosome**	**None**		
	**Cefotaxime (CTX)-30** **μg**	**R**	***bla***_**CTX − M−15**_	**Plasmid**	**None**		
	**Ceftazidime (CAZ)-30** **μg**	**R**	***bla*_OXA − 1_**	**Plasmid**	**None**		
	**Ceftriaxone (CRO)-30** **μg**	**R**					
	**Cefuroxime (CXM)-30** **μg**	**R**					
	**Piperacillin/ Tazobactam (TZP)-110** **μg**	**R**					
**Amphenicol**	**Chloramphenicol (C)-30** **μg**	**R**	***catB3***	**Plasmid**			
**Fluoroquinolones**	**Ciprofloxacin (CIP)-5** **μg**	**R**	**PMQRs:** ***oqxA, oqxB***	**Plasmid**			
	**Nalidixic Acid (NA)-30** **μg**	**R**	**QRDR:** ***gyrA*****: S83I**, ***parC*****: S80I**				
**Polymyxins**	**Colistin (CT)-10** **μg**	**S (13 mm)**				
**Folate Pathway Antagonists**	**Trimethoprim-sulfamethoxazole (SXT)-1.25/23.75** **μg**	**R**	***dfrA14 sul2***	**Plasmid**			
**Tetracyclines**	**Doxycycline (DO)-30** **μg**	**R**	***tet(D)***	**Plasmid**			
**Fosfomycin**	**Fosfomycin (FOS)-50** **μg**	**R**	***fosA***	**Chromosome**			
**Carbapenems**	**Imipenem (IPM)-10** **μg**	**S (23 mm)**				
	**Meropenem (MEM)-10** **μg**	**S (27.9 mm)**				
**Glycylcycline**	**Tigecycline (TGC)-15** **μg**	**R (8 mm)**	***acrA, acrB*** (**Efflux pump** AcrAB)				

**R**, Resistance; **S**, Susceptible.

The *K. aerogenes* (RD99) strain was further subjected to bacterial whole-genome sequencing (WGS) using an Illumina NextSeq 500 platform at icddr,b using an established protocol (28, 29). The raw genome sequence data is publicly available at GenBank with accession number: JAQQRH000000000. The quality control, assembly of sequenced data into draft genomes and annotation were performed using established bioinformatic pipelines (28–30). The FastANI tool was used for taxonomy identification, and *K. aerogenes* was confirmed with a 98.81 Average Nucleotide Identity (ANI) value (31).

The *in-silico* analysis predicted sequence types (ST) and clonal complex using the PubMLST database (https://pubmlst.org/kaerogenes/), capsule polysaccharides using the Kaptive database (32), and plasmid replicons using Plasmid Finder 2.1 (33). The virulence genes were identified using Virulence Factor Database (VFDB) (34). The resistance genes were identified by ABRicate tool v1.0.1 (https://github.com/tseemann/abricate) (35) using Resfinder (36), and NCBI AMR Finder (37) database. Mobile genetic elements using Mobile Element Finder v1.0.3 (38), point mutations using PointFinder software (39), prophage using PHASTER (40), and integrons using IntegronFinder 2.0 (41). Default parameters were applied for all software unless otherwise mentioned. We aligned six *K. aerogenes* isolates (including RD99), 15 publicly available *Klebsiella* species, and 5 *Enterobacter* species genomes with *K. pneumoniae* ATCC 25955 as reference genome using Snippy v4.4.0 (42) and Gubbins v3.2 5 (43). A phylogenetic tree was constructed using RaxML v8.2.12, employing the Generalized Time Reversible substitution model and the GAMMA distribution for rate heterogeneity (44). The tree was visualized using iTOL (45).

The *K. aerogenes* strain (RD99) genome sequence is 5,490,860 bp with a GC content of 54.8%. It comprises 73 contigs, having 5,348 genes, of which 5,224 coding DNA sequences (CDSs) and 124 RNAs. *In silico* profiling revealed that RD99 is a *K. aerogenes strain and* belonged to ST4 and the CC1 clonal complex group. More than 60% (108/175) of the reference virulence genes were detected in the RD99 strain. It harbored the *fim*A-K operon, involved in adherence to human mucosal or epithelial surfaces, *iutA, basG, ent, fep*, and *iro*, which encodes aerobactin, acinetobactin, enterobactin and salmochelin siderophores, *rcsAB*, regulators of mucoid phenotype A. We also found *SenB*, an intrinsic gene encoding toxin (Table 4).

**Table 4:** Genome based virulence gene screening of *Klebsiella aerogenes* (strain RD99) isolated from Dhaka, Bangladesh.

**Virulence categories**	**Virulence factors**	**Related genes**
Adherence	Type 3 fimbriae	*mrkH, mrkI*
	Type I fimbriae	*fimA, fimB, fimC, fimD, fimE, fimF, fimG, fimH, fimI, fimK*
	CFA/I fimbriae (Escherichia)	*cfaB*
	Hemorrhagic E. coli pilus (HCP) (Escherichia)	*hofC*
Antiphagocytosis	Capsular polysaccharide	*wbjD/wecB*
Efflux pump	AcrAB	*acrA, acrB*
Iron uptake	Aerobactin	*iutA*
	Ent siderophore	*entA, entB, entC, entE, entF, entS, fepA, fepB, fepC, fepD, fepG, fes*
	Salmochelin	*IroB, iroC, iroD, iroE, iroN*
	Acinetobactin	*basG*
Regulation	RcsAB	*rcsA, rcsB*
Secretion system	T6SS-I	*clpV/tssH, hcp/tssD, impA/tssA, sciN/tssJ, tssF, tssG, vasE/tssK, vgrG/tssI, vipA/tssB, vipB/tssC*
	T6SS-II	*clpV, dotU, icmF, ompA*
	T6SS-III	*impA, impF*
	Flagella (cluster I) (Yersinia)	*flgA, flgB, flgC, flgD, flgE, flgF, flgG, flgH, flgI, flgJ, flgK, flgL, flgM, flgN, flhA, flhB, flhC, flhD, flhE, fliA, fliE, fliF, fliG, fliH, fliI, fliJ, fliL, fliM, fliN, fliP, fliQ, fliR, fliS, fliT*,
	Hcp secretion island-1 encoded type VI secretion system (H-T6SS) (Pseudomonas)	*clpV1, hcp1*
Toxin	Enterotoxin SenB/TieB	*senB*
	Heat-stable cytotonic enterotoxin	*ast*
Fimbrial adherence determinants	Bcf	*bcfB, bcfD, bcfE, bcfF, bcfG*
	Stc	*stcB, stcC*
Invasion	Flagella	*cheB, cheR, cheW, cheY, cheZ, motA*
Magnesium uptake	Mg2+ transport	*mgtB*
Motility	Flagella	*flaA, motB*

A total of 10 prophage elements were detected, including 3 intact, 5 incomplete and 2 questionable phages and a Class 1 integron was identified. The strain RD99 harbored 17 antibiotic resistance genes that included aminoglycosides resistance genes *aph(3*′*)-Ib, aph(6)*-Id, *aac(3)-Ile, aac(6*′*)-Ib-cr*, β-Lactams resistance genes, *bla*_TEM − 1B_, *bla*_CTX − M−15_, *bla*_OXA − 1_, *bla*_ampC_, folate pathway antagonists resistance genes, *dfrA14, sul2*, plasmid-mediated fluoroquinolones resistance genes, *oqxA, oqxB*, amphenicol resistance gene *catB3*, fosfomycin resistance gene *fosA*, and tetracyclines resistance gene *tet*(D). The efflux pumps AcrAB-associated genes *acrA* and *acrB* were also detected in this strain that would mediate tigecycline resistance. The genes *bla*ampC and *fosA* were located on the chromosome. Other resistance genes were located on plasmids, including *bla*_TEM − 1B_, *bla*_CTX − M−15_, and *bla*_OXA − 1_. We also identified the TN2 transposon element linked with the *bla*_TEM − 1B_ gene. In addition, we identified amino acid substitutions at codon positions S83I in *gyrA* and S80I in *parC*. We also found mutations in *ompK36* gene. No mutations were detected in *ompK35* and *ompK37* genes. In agreement with the phenotypic results, we did not detect any genes conferring resistance to polymyxins and carbapenem antibiotics. We identified five plasmid replicons in RD99 genome, which were Col440I, IncFIB(K), IncFII(K), IncN, IncR (Table 2). The strain RD99 demonstrated OL102 and KL186 antigens.

We conveyed the results of the above analysis, particularly its phenotypic and genotypic features, to the physician. Based on this, the therapy course was changed to meropenem (500 mg/8 h) for 14 days. After 8 days of effective antibiotic treatment and appropriate management of diabetes, the patient became afebrile with no complaints of dysuria and abdominal pain. She was discharged from the hospital and advised to complete the course of antibiotics to avoid the recurrence of the infection. After 14^th^ day the patient recovered completely and demonstrated a negative urine and blood culture. A monthly urine culture was advised for 3 months (one urine CS/month) to assess the efficacy of treatment and the recurrence of infection. All monthly follow-ups (for 3 months) of urine culture and sensitivity tests were negative, and the patient did not experience infection recurrence.

## 3. Discussion

**Figure 1:**
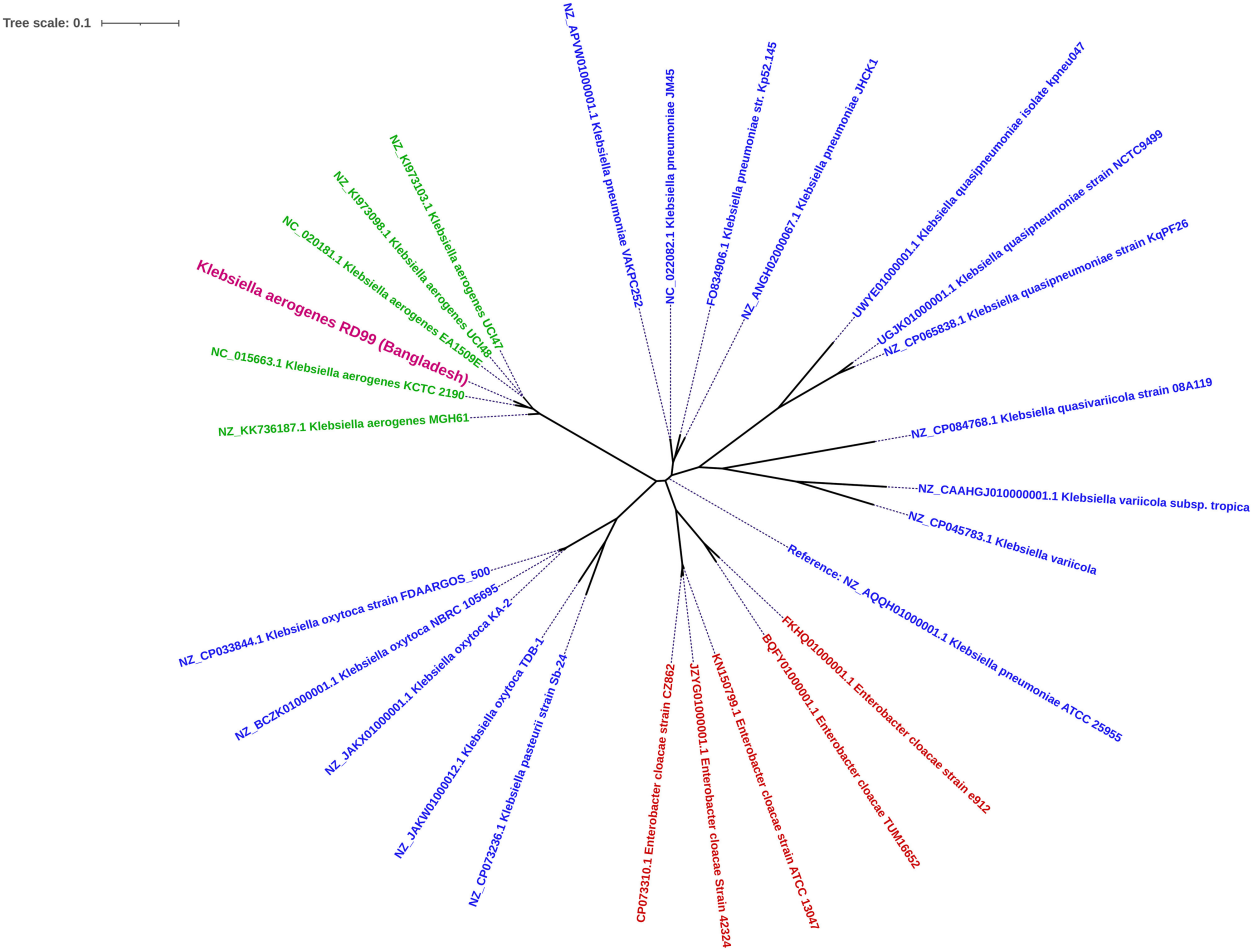
Core-genome SNP-based maximum likelihood phylogenetic tree illustrating the homology between the genome of *Klebsiella aerogenes* RD99 (Violet Color) and other reported genome sequences of *Klebsiella aerogenes* retrieved from NCBI. *klebsiella aerogenes* (Green color) and *Enterobacter* spp (Red color).

*K. aerogenes*, previously known as *Enterobacter aerogenes*, is phylogenetically closer to *Klebsiella* species than *Enterobacter* species (10). *K. aerogenes* is often misidentified as *K. pneumoniae* due to delayed expression of certain phenotypic characteristics (46). This report identified a *K. aerogenes* strain RD99, with delayed motility and ornithine decarboxylase-positivity. Misidentification is also a concern while utilizing automated technologies like Vitek2 and Phoenix (46). Therefore, we carried out bacterial WGS for accurate taxonomic identification. The resulting data revealed that RD99 is 98% similar to the *K. aerogenes* KCTC 2190 type strain. In a phylogenetic analysis, all *K. aerogenes* genomes analyzed belonged to the same clade. Our analysis confirmed that the *Enterobacter aerogenes* genomes were more closely related to *Klebsiella* species than *Enterobacter* species (Figure 1). Based on this study, we propose that the WGS and 16S rRNA gene sequencing techniques (21) may be utilized as an alternative to phenotypic techniques for identifying *K. aerogenes* if confusion arises while employing sophisticated bacteriological identification methods such as API 20E, Vitek2, and Phoenix.

During the consultation, the subject denied any recent history of hospital stays; therefore, she most likely had a community-acquired UTI. However, community-acquired *Klebsiella aerogenes* infection cases are rare, and most reported cases were associated with hospital-acquired infections (12). The patient professed that she was involved with household chores and may have come into contact with environmental sources like contaminated water. *K. aerogenes* is ubiquitous in the environment and human gastrointestinal tract (47). Moreover, a study on the bacteriological quality of drinking water samples across Bangladesh identified *K. aerogenes* from tube wells, a common drinking water source in Bangladesh (48). In addition, poor sanitation practices, such as open defecation, might contribute to spreading germs, resulting in UTIs. Diabetes mellitus has been recognized as a predisposing factor for UTIs. Particularly, those women suffering from T2D are more susceptible to UTIs (49–51). In the case presented here, T2D could be a reason for the serious complications and long recovery from UTI.

The WGS analysis of strain RD99 revealed a similar virulence pattern as reported for earlier *K. aerogenes* (52). The *fim* gene cluster was identified in our strain, which produces Type 1 fimbriae and the adhesive subunit *FimH*. These are key virulence factors responsible for urinary tract infection and biofilm formation (53). The identified Type 3 fimbriae would be involved in adhesion to human tissue structures and biofilm formation, particularly in immunocompromised catheterised patients (54). Siderophores such as aerobactin, acinetobactin, enterobactin, and salmochelin were identified as critical for bacterial virulence (52). Capsular and mucopolysaccharides also play a decisive role in the pathogenesis of *K. aerogenes*, promoting successful establishment of infection by resisting phagocytosis (55). This study also identified the T6SS gene cluster that produces a functioning system that manipulates host cells during pathogenesis and kills competing bacteria (56). The three intact bacteriophages identified in our strain are *Klebsiella* phage ST16-OXA48phi5.4, *Enterobacteria* phage mEp237 and *Escherichia* phage phiV10. Since most *Escherichia* phage phiV10 is commonly found in *E. coli* O157:H7 strain (57), its presence in *K. aerogenes* may indicate a horizontal gene transfer (HGT) event.

It is difficult for clinicians to select the appropriate antimicrobial therapy to treat infections in older patients with co-morbidities like T2D, and the empirical therapy might not be fully effective (16). *Klebsiella* infections develop rapidly, causing multiple organ failures and death. The selection of effective antibiotics with the right dose and course is critical for disease management in older patients to minimize the adverse effects of drugs. Thus, we have evaluated the phenotypic and genotypic antimicrobial resistance features of strain RD99 to initiate antimicrobial therapy. The strain RD99 was classified as XDR using established criteria (58). We observed that the strain RD99 was resistant to most antibiotics with corresponding antibiotic resistance genes, including aminoglycosides, β lactams, amphenicol, fluoroquinolones, and folate pathway antagonists, tetracyclines, phosphonic and glycylcycline antibiotics. However, it was sensitive to carbapenems and polymyxin with no corresponding resistance genes.

Moreover, an inducible chromosomal *bla*_AmpC_ ß-lactamase gene was detected in the RD99 isolate. The high levels of resistance to cephalosporins, even in the presence of a ß-lactamase inhibitor (59), could be due to the *bla*_AmpC_ ß-lactamase gene. Most antibiotic resistance genes found in strain RD99, including *bla*_TEM − 1B_, *bla*_CTX − M−15_, *bla*_OXA − 1_ were found on plasmids, except *bla*_ampC_ and *fosA* genes located on the chromosome. The gene *acrAB* encoding multidrug efflux pump is associated with reduced susceptibility to tigecycline (60). The antibiotic sensitivity tests and antibiotic resistance determinants suggested that the strain RD99 was sensitive to carbapenems.

Studies have shown that the presence of the ompK36 mutation in *Klebsiella aerogenes* is associated with increased resistance to carbapenems, a critical class of antibiotics used to treat serious infections caused by gram-negative bacteria (52). However, in the present study, drug sensitivity test revealed that *K. aerogenes* isolates were all sensitive to carbapenem antibiotics. We did not choose imipenem for an anti-infection treatment because the size of its inhibition zone was just close to threshold, this could be due to ompK36 mutation observed. However, meropenem produced a larger inhibition zone (Table 2). Therefore, meropenem was selected for the management of UTI of the case presented.

In conclusion, the identification of *K. aerogenes* RD99 strain, which had a delayed motility activity and ornithine decarboxylase-positivity, was confirmed through WGS, revealing that this technique may be used as an alternative to phenotypic methods. The patient presented with a community-acquired urinary tract infection, possibly due to poor sanitation practices, contaminated water, and medical conditions such as T2D. Clinicians should initiate an appropriate antimicrobial therapy to treat such infections, as such pathogens are associated with extensive AMR and virulence profiles. Moreover, the strain RD99 was classified as XDR and was resistant to almost all classes of antibiotics. To our knowledge, this is the first in-depth whole-genome-based characterization of *K. aerogenes* isolated from a clinical urine sample in Bangladesh.

## Data availability statement

The datasets presented in this study can be found in online repositories. The names of the repository/repositories and accession number(s) can be found at: https://www.ncbi.nlm.nih.gov/genbank/, JAQQRH000000000.

## Ethics statement

The patient provided written informed consent for the publishing of this case report.

## Author contributions

RM conceptualized the study, performed all *in vitro* tests, carried out whole genome sequencing and drafted the manuscript. AH contributed to discussions and manuscript writing. BB was responsible for obtaining the patient's consent and case history. BB and RM performed bioinformatics analysis, interpreted the data, and generated tables and figure. JP, SC, TC, and DM participated in the discussions and reviewed the manuscript. DM supervised the research. The final paper has been reviewed and approved by all authors.
